# Effect of a community‐based child health counselling intervention on health‐seeking behaviours, complementary feeding and nutritional condition among children aged 6–23 months in rural China: A pre‐ and post‐comparison study

**DOI:** 10.1111/mcn.13289

**Published:** 2021-11-23

**Authors:** Shiyi Yao, Shuyue Xiao, Xi Jin, Mei Xiong, Jing Peng, Lijun Jian, Yongli Mei, Yuehua Huang, Haiqun Zhou, Tao Xu

**Affiliations:** ^1^ Child Health Care Department, National Center for Women and Children's Health Chinese Center for Disease Control and Prevention Beijing China; ^2^ Division of Program Management Liangshan Yi Autonomous Prefecture Hospital for Women and Children's Health Sichuan China; ^3^ Child Health Care Department Butuo County Hospital for Women and Children's Health Sichuan China; ^4^ Division of Program Management Meigu County Hospital for Women and Children's Health Sichuan China; ^5^ Child Health Care Department Yuexi County Hospital for Women and Children's Health Sichuan China; ^6^ Child Health Care Department Zhaojue County Hospital for Women and Children's Health Sichuan China

**Keywords:** children, complementary feeding, counselling, growth, nutrition, nutritional interventions

## Abstract

In China, the prevalence of undernutrition among children under 5 years of age has declined significantly during recent decades. However, noticeable gaps exist between rural and urban areas. Since 2012, a government‐funded nutrition programme, Ying Yang Bao (YYB; soybean powder‐based iron‐rich supplement) programme, has been implemented in poor rural areas to decrease the risk of developing anaemia among children aged 6–23 months, but there are still inadequate health care awareness, feeding knowledge and skills among caregivers. From June 2018 to December 2020, a child health counselling intervention was delivered through a home visit based on the YYB programme in Liangshan. Child health messages were given by trained village child health assistants while distributing YYB. Surveys were conducted before and after the intervention to analyse changes in child health check‐up frequency, complementary feeding practice and prevalence of undernutrition. After the intervention, the proportion of children who had regular health check‐ups, who were vaccinated and who met the minimum YYB consumption significantly increased from 26.0%, 81.6%, and 67.8% to 59.7%, 95.0%, and 79.2%. Increased rates of IYCF indicators (introduction of solid, semisolid, or soft foods, minimum dietary diversity and consumption of iron‐rich or iron‐fortified foods) were observed after the intervention. The prevalence of stunting, underweight, wasting, and anaemia significantly decreased from 26.3% to 10.8%, 13.4% to 8.7%, 14.0% to 10.5%, and 52.1% to 43.9%. This intervention can be well integrated into the YYB programme with less additional resources. Children in resource‐limited areas will benefit more from a comprehensive nutritional package, including food supplements and child health education.

## Key messages

- Current evidence suggests there is a decrease in the prevalence of undernutrition among children under 5 years of age in China during recent decades. However, imbalanced development exists between rural and urban areas.
- Government‐funded Ying Yang Bao (YYB) programme has been implemented in poor rural areas across China covering over 800 counties, to decrease the risk of developing anaemia among children aged 6–23 months.
- After a child health counselling intervention jointly implemented with YYB programme, the prevalence of stunting, underweight, wasting, and anaemia among children decreased significantly.
- Child health counselling intervention could be well integrated into the ongoing YYB programme to reach resource‐limited areas on a large scale.

## Graphical abstract

This study compared the results of two cross‐sectional surveys before and after the community‐based child health counselling intervention. After the intervention, nutritional conditions among children aged 6–23 months improved with the reduced prevalence of stunting, undernutrition, wasting, and anaemia. Being integrated into the ongoing Ying Yang Bao (YYB) programme, this intervention could reach resource‐limited areas on a large scale with minimum resources.

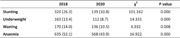

## 1 INTRODUCTION

Childhood undernutrition is a major global public health challenge. According to the World Health Organization (2021) estimation, 149.2 million children under 5 years of age were suffering from stunting, 85.4 million from underweight and 45.4 million from wasting in 2020. Nutritional deficiency is also one of the most common causes of anaemia affecting 42% of children under 5 years of age (World Health Organization, 2020). Worldwide, undernutrition was the cause of death for 3.1 million children under 5 years of age, accounting for 45% of deaths in all children under 5 years of age in 2011 (Black et al., 2013). Also, malnourished children have a higher risk of death from diarrhoea, pneumonia, and other infectious diseases. In addition to the increased risk of child morbidity, early childhood undernutrition has long‐term negative effects on adolescent and adult health. Growing evidence shows that suboptimal childhood nutrition may exacerbate inequity and poverty by lowering educational attainment and reducing adult productivity, thus leading to the intergenerational transmission of poverty (Black et al., 2013).

In China, the prevalence of stunting, underweight, and wasting among children under 5 years of age has declined significantly during the decade between 2002 and 2013 (Chang & Wang, 2016). However, there are still noticeable gaps between rural and urban areas. Chang and Wang (2016) reported in the National Nutrition and Health Survey in 2013, among children under 5 years of age, 19.0% in poor rural areas and 4.2% in urban areas were stunted; 5.1% and 1.7% were underweight; 2.7% and 1.5% were wasted. Anaemia also remains a problem in China, particularly in rural areas: 28% of rural children aged 6–11 months and 21% aged 12–23 months are estimated to be anaemic (Li et al., 2019).

To build a more prosperous society, health poverty alleviation has been launched as a national strategy to accelerate the development of impoverished areas. Since 2012, a government‐funded infant nutrition improvement programme has been implemented in poor rural areas across China, as part of the national poverty alleviation strategy (Xu et al., 2019). This programme aims to decrease the risk of developing anaemia among children by distributing soybean powder‐based and iron‐rich food supplements, also known as Ying Yang Bao (YYB), to children aged 6–23 months free of charge. It is recommended for children to consume no less than four sachets of YYB (12 g/sachet) every week (preferably one sachet/day). YYB programme has reached a large number of children to improve their nutritional conditions so far (Xu et al., 2019). In Liangshan Yi autonomous prefecture (Liangshan), one of the most impoverished areas in China, YYB programme has been integrated into primary child health care services. Child health workers at the grassroots level distributed YYB while carrying out routine health service delivery. However, gaps still exist as there are inadequate health care awareness, feeding knowledge and skills among caregivers. A recent study conducted in Liangshan showed that the percentage of caregivers knowing key information related to child nutrition was still below 50% (Yao et al., 2020). This indicated that the gaps exist between senders (health workers) and recipients (caregivers) during the dissemination of health information. How to deliver a comprehensive package of child nutrition services efficiently and effectively in rural settings remains a challenge. Context‐based and need‐oriented educational interventions are needed to reach the caregivers and fill these gaps.

Since July 2017, to accelerate the progress of health poverty alleviation, China's National Health Commission (NHC) cooperated with Bill & Melinda Gates Foundation (BMGF) to implement ‘Child Nutrition and Health Program’ in 10 townships of the four poorest counties in Liangshan. This program aims to explore the effective approach to deliver child health service that fits into impoverished areas, such as Liangshan. It is also to explore the feasibility and effectiveness of a counselling intervention integrated into YYB distribution and to provide evidence‐based information to other YYB programme areas. From June 2018 to December 2020, a child health counselling intervention, targeting caregivers with children aged 6–23 months, was delivered through home visits to improve health check‐up awareness and appropriate complementary feeding skills, mostly to improve child nutrition and health. Community‐based counselling was conducted by trained village child health assistants (CHAs) while distributing YYB. This study compares the results before and after the intervention to analyse changes in child health check‐up frequency and complementary feeding practice, as well as the prevalence of stunting, underweight, wasting, and anaemia.

## 2 METHODS

### 2.1 Study setting

Liangshan, located in the southwest of Sichuan province, is a mountainous multiminority region and the biggest settlement of Yi minority. Affected by multiple socioeconomic factors, Liangshan is one of the most impoverished areas in China. The present study was conducted in 10 selected poorest townships of four counties. The total population of 10 townships was around 127,000. There were around 4000 children aged 6–23 months, accounting for 3.1% of the whole population. Each county has a county‐level hospital for women and children's health, and each township has a township health care center. According to China's primary health care policy, these health facilities provide a package of 14 basic health care services free of charge for community residents, including vaccination and regular health check‐up for children (at 3, 6, 8, 12, 18, 24, 30 months of age and 3, 4, 5, 6 years of age) (National Health Commission, 2012).

### 2.2 Study population and sampling method

This is a precomparison and postcomparison study, comparing data before and after the implementation of the intervention. Before the intervention, a cross‐sectional survey using a stratified random sampling method was conducted from April to July in 2018. Thirty percent of children aged 6–23 months in each township were randomly selected using child registration sheets stratified into three age groups as 6–11 months, 12–17 months, and 18–23 months. After the intervention, from June to July in 2020, participants were recruited using the same method. A total of 1218 and 1293 child‐caregiver pairs participated at pre‐ and post‐study, respectively.

### 2.3 Intervention

Before the initiation of child health counselling intervention, 127 village CHAs were recruited and each village had at least one village CHA. These part‐time village CHAs had at least a middle school education and most of them had health‐related education backgrounds. They were then trained by medical staff from township health care centres. The training courses included growth monitoring, complementary feeding and related child health care information. From June 2018 to December 2020, based on the implementation structure of YYB programme, village CHAs distributed 30 sachets of YYB to each child aged 6–23 months every month through home visits. During home visits, village CHAs marked the child's height and weight on the growth chart in the child health record, and reminded caregivers when children reached the age for a regular health check‐up. They also gave age‐appropriate complementary feeding suggestions. Specific messages included (1) continued breastfeeding after 6 months of age and up to 2 years of age or beyond; (2) starting to add complementary food no later than 6 months; (3) food texture, diversity, and meal frequency at different ages; (4) one YYB every day (no less than four sachets/week). Every month, medical staff from the township health centre provided regular child health training for village CHAs while implementing the quality control activities. The village CHAs could get corresponding subsidies based on their number of home visits and the quality of counselling.

### 2.4 Data collection

The pre‐ and post‐data collection were conducted using the same method by trained township medical staff. Caregiver and child were invited to the township health centre for a face‐to‐face interview. During the interview, the medical staff communicated with the caregiver in a local dialect while completing the questionnaire in mandarin accordingly. The questionnaire collected information on socio‐demographic characteristics of parents, frequency of health check‐ups, YYB consumption, diversity and frequency of meals in the past 24 h. The child's physical measurements (height and weight) and haemoglobin concentrations were obtained from the child health record.

### 2.5 Study indicators

*Stunting, underweight, and wasting* are defined as the *z*‐scores of length‐for‐age, weight‐for‐age and weight‐for‐length, respectively, being more than two *SD*s below the median of reference population using WHO child growth standards (World Health Organization, 2006).

*Anaemia* is defined as haemoglobin level being less than 110 g/L according to WHO recommendations. Adjustments to the measured haemoglobin concentrations were made according to the altitude (World Health Organization, 2011).

*Breastfed children*: Children who received breast milk during the previous day.

*Infant and young child feeding (IYCF) indicators*: Among eight core WHO IYCF indicators, six indicators concerning children aged 6–23 months were used in this study (World Health Organization, 2008).

*Continued breastfeeding at 1 year*: Proportion of children 12–15 months of age who are fed breast milk.

*Introduction of solid, semisolid or soft foods*: Proportion of infants 6–8 months of age who receive solid, semisolid, or soft foods.

*Minimum dietary diversity (MDD)*: Proportion of children 6–23 months of age who receive foods from four or more food groups.

*Minimum meal frequency (MMF)*: Proportion of breastfed and nonbreastfed children 6–23 months of age who receive solid, semisolid or soft foods (but also including milk feeds for nonbreastfed children) the minimum number of times or more.

*Minimum acceptable diet (MAD)*: Proportion of children 6–23 months of age who receive a minimum acceptable diet (apart from breast milk). For breastfed children, a minimum acceptable diet means to have at least the MDD and the MMF. For nonbreastfed children, it means to receive at least teo milk feedings and have at least the MDD not including milk feeds and the MMF.

Consumption of iron‐rich or iron‐fortified foods: Proportion of children 6–23 months of age who receive an iron‐rich food or iron‐fortified food that is specially designed for infants and young children, or that is fortified in the home. In this study, iron‐rich or iron‐fortified food specifically refers to YYB.

### 2.6 Data analysis

All statistical analyses were conducted using SPSS software (version 23; IBM). *χ* ^2^ analysis was performed to determine the differences in socio‐demographic characteristics and changes in regular health check‐ups, YYB consumption, IYCF indicators and nutritional conditions (stunting, underweight, wasting, and anaemia) before and after the intervention. Statistical significance was defined as *p* < 0.05.

## 3 RESULTS

### 3.1 Socio‐demographic characteristics

Characteristics of children and their parents are presented in Table 1. In 2018, a total of 1218 child‐caregiver pairs participated in the study and 1293 child‐caregiver pairs participated in 2020. No significant difference was observed concerning child gender, age group, preterm, low birthweight, and breastfeeding status (*p* > 0.05).

**Table 1:** Socio‐demographic characteristics of children and their parents in 2018 and 2020 (%)

	2018 (*n* = 1218)	2020 (*n* = 1293)	*χ* ^2^	*p* value
Gender (child)			3.563	0.059
Female	595 (48.9)	583 (45.1)		
Male	623 (51.1)	710 (54.9)		
Age group (child)			5.030	0.081
6–11 month	493 (40.5)	530 (41.0)		
12–17 month	401 (32.9)	378 (29.2)		
18–23 month	324 (26.6)	385 (29.8)		
Preterm (child)			0.259	0.611
No	1166 (99.1)	1118 (98.9)		
Yes	11 (0.9)	13 (1.1)		
Birthweight (child)			0.032	0.857
≥2500 g	1180 (97.4)	1053 (97.3)		
<2500 g	31 (2.6)	29 (2.7)		
Breastfed (child)			3.288	0.070
No	342 (28.1)	397 (31.4)		
Yes	875 (71.9)	866 (68.6)		
Education (mother)			4.437	0.035
Primary and below	995 (91.4)	1137 (88.8)		
Secondary and above	94 (8.6)	144 (11.2)		
Occupation (mother)			26.472	0.000
Housework	667 (61.2)	652 (50.9)		
Farmer	375 (34.4)	544 (42.5)		
Other	47 (4.3)	84 (6.6)		
Education (father)			19.545	0.000
Primary and below	956 (86.8)	1020 (79.9)		
Secondary and above	146 (13.2)	256 (20.1)		
Occupation (father)			416.245	0.000
Housework	593 (53.9)	275 (21.6)		
Farmer	447 (40.6)	548 (43.0)		
Other	60 (5.5)	452 (35.5)		

### 3.2 Child health‐seeking behaviour

Before the intervention, 273 (26.0%) children had regular health check‐ups in the previous year, 981 (81.6%) were vaccinated, and 540 (67.8%) had at least four YYB sachets per week (Table 2). Among the caregivers, 75.3% of them reported having consulted with health workers on child health care issues, such as growth and complementary feeding. After the intervention, the proportion of children who had regular check‐ups, who were vaccinated and who met the minimum YYB consumption significantly increased to 59.7%, 95.0%, and 79.2%, respectively. There was also a significant improvement in caregivers consulting with health workers (Table 2).

**Table 2:** Child health‐seeking behaviour in 2018 and 2020 (%)

	2018	2020	*χ* ^2^	*p* value
Regular health check‐ups in the previous year	273 (26.0)	635 (59.7)	244.152	0.000
Ever vaccinated	981 (81.6)	1223 (95.0)	110.285	0.000
Caregivers consulting with health workers	733 (75.3)	1228 (97.1)	240.895	0.000
No less than 4 YYB sachets/week	540 (67.8)	996 (79.2)	33.600	0.000

### 3.3 IYCF indicators

The continued breastfeeding rate at 1 year was slightly lower in 2020 compared to 2018 (69.0% and 69.5%) (Table 3). Increased rates of introduction of solid, semisolid, or soft foods (83.1% and 84.1%), MDD (49.5% and 51.4%) and consumption of iron‐rich or iron‐fortified foods (55.0% and 84.5%) were observed from 2018 to 2020, while only the increase of consumption of iron‐rich or iron‐fortified foods was statistically significant. In 2020, 65.1% of children had achieved MMF and 26.9% achieved MAD, no data were collected in 2018. Nonbreastfed children were more likely to achieve MMF (69.7% and 62.7%), while breastfed children were more likely to achieve MAD (31.2% and 18.6%) (Table 3).

**Table 3:** IYCF indicators in 2018 and 2020 (%)

	2018	2020	χ^2^	*p* value
Continued breastfeeding at 1 year	166 (69.5)	158 (69.0)	0.012	0.914
Introduction of solid, semisolid or soft foods	207 (83.1)	191 (84.1)	0.088	0.767
Minimum dietary diversity (MDD)	598 (49.5)	644 (51.4)	0.844	0.358
Minimum meal frequency (MMF)	–	677 (65.1)		
Breastfed children	–	428 (62.7)		
Nonbreastfed children	–	249 (69.7)		
Minimum acceptable diet (MAD)	–	276 (26.9)		
Breastfed children	–	212 (31.2)		
Nonbreastfed children	–	64 (18.6)		
Consumption of iron‐rich or iron‐fortified foods	516 (55.0)	1061 (84.5)	232.655	0.000

### 3.4 Nutritional conditions

Prevalence of stunting, underweight, wasting and anaemia were significantly lower in 2020, decreasing from 26.3% to 10.8%, 13.4% to 8.7%, 14.0% to 10.5% and 52.1% to 43.9%, respectively (Table 4).

**Table 4:** Prevalence of stunting, underweight, wasting and anaemia in 2018 and 2020 (%)

	2018	2020	χ^2^	*p* value
Stunting	320 (26.3)	139 (10.8)	101.162	0.000
Underweight	163 (13.4)	112 (8.7)	14.331	0.000
Wasting	170 (14.0)	136 (10.5)	6.932	0.008
Anaemia	635 (52.1)	568 (43.9)	16.922	0.000

## 4 DISCUSSION

This study compared the results of two cross‐sectional surveys before and after the community‐based child health counselling intervention. The results showed that children had better adherence to regular health check‐ups and YYB consumption after the intervention. There was a minor improvement in achieving the IYCF indicators. Nutritional conditions among children aged 6–23 months also improved with the reduced prevalence of stunting, undernutrition, wasting, and anaemia.

Our results showed that the child health counselling intervention carried out by village CHAs significantly increased the adherence to regular health check‐ups among children. According to the national primary health care policy, every child below 7 years old is to be brought to community health care centres in urban areas and township health care centres in rural areas at specific intervals for growth monitoring, disease screening, vaccination and health counselling (National Health Commission, 2012). This Systematic Health Care for Children (SHCC) is provided free of charge. Although the national regular health check‐up coverage of children under 3 years of age has reached over 90% in 2018, there are still some obstacles hindering children in poor rural areas to receive these services (National Health Commission, 2020). As the baseline data revealed, only 26.0% of children had regular health check‐ups in 2018, which was much lower than the national level, also lower than the average of the most impoverished areas in Sichuan province (37.5%) (Guan et al., 2020). The child health counselling on the benefit of growth monitoring and complementary feeding helped caregivers to establish and emphasise the inner connection between inappropriate feeding, delayed growth, and nutritional conditions. Therefore, it improved parents' awareness and stimulated their needs to seek child health care services. Baseline data also indicated that parents' acceptance of vaccination was high. So township health centres optimised the procedure for children to have health check‐ups before vaccination at every visit. As a result, it increased the regular health check‐up rate from 26.0% to 59.7% and the vaccination rate also increased from 81.6% to 95.0%.

After the intervention, the nutritional conditions of children aged 6–23 months in Liangshan improved significantly. The prevalence of stunting (10.8%) in 2020 was lower than that in other poor areas in west China, such as Chongqing (13.7%) and Yunnan (11.1%), and even below the national average level among poor rural areas (19.0%) (Chang & Wang, 2016; Chen et al., 2020; Jiang et al., 2017). Prevalence of underweight (8.7%) and wasting (10.5%) also decreased significantly in 2020, but still higher than the national average level (5.1% and 2.7%) (Chang & Wang, 2016). The postintervention prevalence of anaemia (43.9%) was lower compared to other poor areas in west China (Chen et al., 2017; Jiang et al., 2017; Zhang et al., 2016; Zhao et al., 2017). It was comparable to that in other Asian countries, such as Bangladesh (43.1%) and Bhutan (44.7%), but higher than that in Indonesia (38.4%), Malaysia (24.6%), and Thailand (24.9%) (WHO, 2020). These findings not only proved the effectiveness of the intervention but also emphasised the need for sustainable investment in infant feeding promotion programmes in these areas.

One of the expected outputs of health counselling was to improve the feeding practices, therefore to meet IYCF indicators. However, unlike the significant improvement in nutritional conditions, our results only detected a minor improvement in IYCF indicators: the continued breastfeeding rate at 1 year decreased slightly, the proportion of children who had introduced solid, semisolid, or soft foods and met MDD increased with no statistical significance. Only the proportion of children who consumed iron‐rich or iron‐fortified foods increased significantly. Previous studies found mixed results concerning the association between IYCF indicators and anthropometric outcomes. Some reported a lack of association, and the authors argued that the indicators might not be sensitive or specific (Bentley et al., 2015; Rakotomanana et al., 2017; Walters et al., 2019). It could also be argued that the 24 h‐recall method did not reflect the whole situation of feeding practices as it only considered meals of the previous day (Jones et al., 2014).

Nationally, 43.3% of children continued breastfeeding at 1 year in 2018, which was higher in the rural area (46.1%) than in urban (40.3%) (National Health Commission, 2021). The median continued breastfeeding period was 12–14 months in west rural China (Liang et al., 2007; Wang et al., 2013). Our findings indicated that Liangshan (69.0%) has a comparatively high continued breastfeeding rate; however, more efforts are still needed nationwide to achieve WHO recommendation of continued breastfeeding at 2 years. Adding complementary foods at the right time is also crucial to infants' health. Our study showed that the majority (84.1%) of children had received complementary food at 6–8 months in Liangshan, which was higher than the national level (60.2%) reported in 2018 (National Health Commission, 2021). Previous studies in other provinces showed that about 1/4 of children aged 1–2 months were introduced complementary food in rural Yunnan (Li & Hu, 2015). In rural areas of Guizhou province, the average age when children first received complementary foods was 3.5 months (Leng et al., 2017). Thus, it should be taken into consideration that the high complementary food introduction rate in Liangshan might indicate the early introduction of complementary food. There were geographic differences in terms of MDD, MMF, and MAD across poor rural west China. In Guizhou province, for example, 68.6%, 40.1%, and 28.1% of children achieved MDD, MMF, and MAD (Zhang et al., 2016). And in Gansu province, there were 64.1%, 70.6%, and 42.2%, respectively (Lu et al., 2020). Variation was also observed in studies conducted in different countries. A review of IYCF in south Asia estimated that MDD, MMF, and MAD were 33.0%, 47.7%, and 20.5%, respectively, with MDD ranging from 15.3% in Bhutan to 88.0% in Sri Lanka (Aguayo, 2017). Our results found that the MDD, MMF, and MAD in Liangshan were 51.4%, 65.1%, and 26.9% in 2020, comparable to the national level (53.7%, 69.1%, and 25.1%, respectively), which still leaves room for improvement (Duan et al., 2018).

As health beliefs were rooted in the long‐established traditions, it will need long‐term interventions to improve feeding practices. It is very important to find a solution to incorporate any intervention into the existing health care system (Zhou et al., 2019). In China and other countries with a well‐established grassroots health care services system, better leveraging of the strength of the existing system is important and more sustainable. This child health counselling intervention was integrated into the ongoing YYB programme. It did not require additional resources and could be delivered with a minimum workload. CHAs as the link between families and township health care centres, delivered the single ‘small’ nutritional package (YYB) and the comprehensive ‘big’ nutritional package (feeding practice counselling plus health check‐up reminder) through frequent home visits. As a government‐funded nationwide programme, YYB programme covers over 800 impoverished counties across 22 prioritised provinces. Thus this study provided a good model to deliver child health counselling services to resource‐limited areas on a large scale.

## 5 LIMITATIONS

This study was subjected to several limitations. First, this study was a pre‐post design. The results might be biased due to secular trends or the introduction of other interventions during the time frame of this study. Second, some socioeconomic characteristics, such as sanitation, household income, and availability of local food were not assessed, which might have an influence on complementary feeding or nutritional conditions. Third, self‐reported information on the frequency of health check‐ups, frequency and diversity of meals might be subjected to recall and social desirability biases and therefore affect the accuracy of the information provided.

## CONFLICT OF INTERESTS

The authors declare that there are no conflict of interests.

## AUTHOR CONTRIBUTIONS

Xi Jin and Tao Xu designed the research study. Shiyi Yao, Tao Xu, Mei Xiong, Jing Peng, Lijun Jian, Yongli Mei, Yuehua Huang, and Haiqun Zhou performed the research. Shiyi Yao, Shuyue Xiao and Tao Xu analysed the data. Shiyi Yao and Tao Xu wrote the paper.

## ETHICS STATEMENT

Ethical clearance was obtained from National Center for Women and Children's health, Chinese Center for Disease Control and Prevention. Participants were involved in the survey on a voluntary basis. Written consent, signed or verified by fingerprint, was obtained from caregivers on behalf of their children.

## Data Availability

The data that support the findings of this study are available from the corresponding author upon reasonable request.
